# Ecological science and tomorrow's world

**DOI:** 10.1098/rstb.2009.0164

**Published:** 2010-01-12

**Authors:** Robert M. May

**Affiliations:** Zoology Department, Oxford University, Oxford OX1 3PS, UK

**Keywords:** biodiversity, extinction rates, population growth, ecological footprints, cooperation

## Abstract

Beginning with an outline of uncertainties about the number of species on Earth today, this paper addresses likely causes and consequences of the manifest acceleration in extinction rates over the past few centuries. The ultimate causes are habitat destruction, alien introductions, overexploitation and climate change. Increases in human numbers and *per capita* impacts underlie all of these. Against a background review of these factors, I conclude with a discussion of the policy implications for equitably proportionate actions—and of the difficulties in achieving them.

It takes a very bold, or perhaps a very foolish, individual to write an essay on their field's big questions, much less on promising approaches that could produce a paradigm shift. One does well to keep in mind some of the spectacularly silly things that have been said in such a context: ‘The time has come to close the book on infectious diseases’ (the US Surgeon General in 1997); ‘Heavier than air flying machines are not possible’ (Lord Kelvin, PRS 1895); ‘There is a world market for fifteen large computers’ (Chairman of IBM 1945).

My field is ecology, broadly defined. And so, boldly or foolishly or otherwise, this essay aims briefly to sketch some areas where recent advances or continuing gaps in ecological science intersect with environmental problems and policy issues.

The essay is organized as follows. First, looking beyond ourselves, I focus on rising rates of species extinctions among plants and other animals: how much of Nature's diversity is known to us; how do recent and likely future extinction rates compare with what we know of the fossil record; how well do we understand the interplay between biological diversity and ecosystem services that humans depend on. Second, I turn briefly to the causes of the rising rates of biological impoverishment. Ultimately, these can be seen as resulting from the increasing magnitude of humanity's ‘ecological footprint’ (EF), itself a combination of more people and more impact per person. So §3 deals with human population growth, and §4 with rising impacts (over the past 150 years, population increased roughly sevenfold, and so has the average *per capita* energy consumption, for an overall 50-fold growth in humanity's EF). The fifth and concluding section indicates some of the policy implications, essentially all of which involve a major problem—arguably evolutionary biology's major unsolved problem—of the occurrence and maintenance of cooperative behaviour in large groups of unrelated humans.

## 1. Extinction rates: past, present, future

According to its website on 20 December 2008, the US Library of Congress has exactly 20 854 810 catalogued books. In our planet's library of life, how many distinct species of plants and animals are known to science? Probably around 1.6 million or so, but this number is uncertain to around 10 per cent. Despite recent advances in coordinating and digitizing information held in many institutions around the world (which taken at face value might suggest a number more like 1.8–1.9 million), there remain major uncertainties caused by synonyms—the same species separately identified, and separately named, in different collections. For the largest single taxonomic group, beetles, estimates suggest roughly 40 per cent of species are known from only one site, some from only one specimen. As older synonyms are being resolved, recent collections are producing new ones. A seminal analysis of this problem by Solow *et al*. (1995) suggests a synonymy rate of around 20 per cent in most invertebrate collections; recent advances are undoubtedly reducing this, but not to zero. We have a long way to go to rival the accuracy of library catalogues (for further discussion see Godfray *et al*. 2008).

The more important question is how many distinct species are currently to be found on Earth? Numbers as high as 100 million and as low as three million have been suggested, and plausible estimates span the range 5–10 million or so (Hammond 1995; May 1999). Bird and mammal species (comprising 1% of all known species, but attracting roughly one-third of taxonomic efforts) are well studied, and new species turn up at the rate of only a few each year. A similar one-third of taxonomic attention is given to plant species (around 20% of the species' total). The vastly greater number of invertebrate animal species—the small things which arguably run the world—attracts the remaining one-third.

Given these facts, it is not surprising that we know even less about the numbers of species to have become extinct over the past few decades. Table 1 (compiled from the IUCN Red Data Book 2004) makes this plain. Although a lot is known about the status of birds, mammals and amphibians, the other vertebrates (reptiles and fish) do less well. This is even more true for plants. And our ignorance about invertebrate animals is emphasized by the fact that apparently only 0.06 per cent of those known to science—never mind the larger number not known—are endangered. But, when re-expressed as a fraction of those evaluated, the number changes to 73 per cent (admittedly, this also reflects the fact that, unlike birds and mammals, invertebrates only get evaluated when someone knows them and is worried about them).

**Table 1.** Species threatened with extinction (IUCN Red Data Book 2004).

taxon	all known species in taxon (% threatened)	fraction threatened for species of evaluated status (%)
vertebrates		
mammals	20	23
birds	12	12
amphibians	31	31
reptiles	4	61
fish	3	26
plants		
dicots	4	74
monocots	1	68
invertebrates		
insects	0.06	73

We can, however, say some relatively precise things about extinction rates, in relation to the average rates seen over the 550 Myr sweep of the fossil record. For the relatively well-studied bird and mammal species, there has been roughly extinction of one species per year over the past century. This estimate, moreover, is very conservative. There are a total of around 14 000 such species. So the typical bird or mammal species has, in effect, in recent years been playing a game of Russian Roulette with a single bullet in a gun of 14 000 chambers. This translates into an average expected species lifetime, before extinction, of around 10^4^ years at current rates, if birds and mammals are typical (which, of course, they might not be). Ten thousand may sound a long time, but it is shorter by a factor of order 10^−2^ to 10^−3^ than the background average lifespan of 10^6^ to 10^7^ years seen in the fossil record. That is, recent extinction rates in well-documented groups have run 100–1000 times faster than the average background rates. This is the same acceleration in extinction rates as characterizes the Big Five episodes of mass extinction in the fossil record. And four different approaches to estimating impending rates of extinction suggest further acceleration by a factor 10 or more (for details, see May 1999).

**Figure 1.**
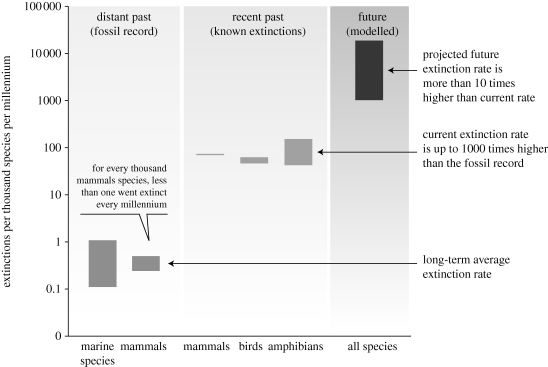
Species' extinction rates, expressed as extinctions per thousand species per millennium. ‘Distant past’ refers to average extinction rates as estimated from the fossil record; ‘recent past’ refers mainly to the past century and ‘future’ estimates are based on a variety of techniques (species–area models; rates at which species are shifting to increasingly more threatened categories and others) and pertain mainly to the next few centuries. For details, see Millennium Ecosystem Assessment (2005).

All this is summarized well in figure 1, taken from the Millennium Ecosystem Assessment (2005), which involved some 1360 scientists from 95 countries. This shows variously estimated extinctions per thousand species per millennium in the ‘distant past’ (average rates, as deduced from the fossil record), ‘recent past’ (1900s) and future (next several centuries).

Setting aside ethical questions about our responsibility to future generations, why should we care about the impending extinction spasm? The Millennium Ecosystem Assessment argues that utilitarian considerations alone should prompt grave concern. This conclusion is based on a comprehensive appraisal of the condition of, and trends in, the world's ecosystems. Ecosystem services are the benefit provided to humans as a result of species' interactions within the system. Some of these services are local (e.g. provision of pollinators for crops), others regional (e.g. flood control or water purification) and yet others global (e.g. climate regulation). In its massive report, the Millennium Ecosystem Assessment identifies 24 categories of such ecosystem services, broadly grouped under three headings: provisioning, regulating and cultural.

Table 2 summarizes these 24 categories of service, along with indications of whether the services are being enhanced or degraded, according to fairly precise criteria (Millennium Ecosystem Assessment 2005). Note that, of the 24 categories of ecosystem services, 15—roughly two-thirds—are being degraded or used unsustainably. Four, of which three involve food production, have been enhanced in the past 50 years. The status of the remaining five is equivocal, as indicated in the table.

**Table 2.** Global status of ecosystem services (Millennium Ecosystem Assessment 2005).

service	status^a^	notes
*provisioning services*		
food		
crops	+	substantial production increase
livestock	+	substantial production increase
capture fisheries	−	declining production due to overharvest
aquaculture	+	substantial production increase
wild foods	−	declining production
fibre		
timber	±	forest loss in some regions, growth in others
cotton, hemp, silk	±	declining production of some fibres, growth in others
wood fuel	−	declining production
genetic resources	−	lost through extinction and crop genetic resource loss
biochemicals, natural medicines, pharmaceuticals	−	lost through extinction, overharvest
fresh water	−	unsustainable use for drinking, industry and irrigation; amount of hydro energy unchanged, but dams increase ability to use that energy
*regulating services*		
air quality regulation	−	decline in ability of atmosphere to cleanse itself
climate regulation		
global	+	net source of carbon sequestration since mid-century
regional and local	−	preponderance of negative impacts
water regulation	±	varies depending on ecosystem change and location
erosion regulation	−	increased soil degradation
water purification and waste treatment	−	declining water quality
disease regulation	±	varies depending on ecosystem change
pest regulation	−	natural control degraded through pesticide use
pollination	−^b^	apparent global decline in abundance of pollinators
natural hazard regulation	−	loss of natural buffers (wetlands, mangroves)
*cultural services*		
spiritual and religious values	−	rapid decline in sacred groves and species
aesthetic values	−	decline in quantity and quality of natural lands
recreation and ecotourism	±	more areas accessible but many degraded

^a^+ means enhanced,−means degraded, in the senses defined in the main text.

^b^The evaluation here is of ‘low to medium certainty’; all other trends are ‘medium to high certainty’.

The way economists conventionally calculate gross domestic product (GDP) takes little or no account of the role of ecosystem services. So an oil tanker going aground, and wreaking havoc on the region's biota, will typically make a positive contribution to conventional GDP (cleanup costs are a plus; environmental damage deemed not assessable). Constanza *et al*. (1997) have attempted to assess the ‘GDP-equivalent’ of the totality of the planet's ecosystem services. Their guesstimate is that such services have a value roughly equal to global GDP as conventionally assessed. Any calculation of this kind is beset with many uncertainties, and some would argue that you simply cannot put a price upon a service that is essential to life. I nevertheless find it helpfully indicative.

One important step in the direction of a more explicit and rigorous characterization of the components of ecosystem services is to develop indicators. It can be argued that ecologists and conservation biologists could learn from economists' long-standing set of common and clear indicators, which despite their recently exposed imperfections can be helpful in tracking and influencing the development of markets.

In principle, such worries about the impacts that loss of species can have on the survival of ecosystems could be counterbalanced by faith that human ingenuity could—if we understood the structure and function of natural ecosystems well enough—be managed to deliver crucial ecosystem services in a biologically impoverished world. Again setting aside the question of whether we would want to live in the world of the cult movie *Bladerunner*, we must ask how close we are to such understanding. Notwithstanding significant recent advances, I think we have still a long way to go (Dunne 2006; May 2006, 2007). But that is another essay.

## 2. Causes of species' extinctions

As reviewed by Diamond (1989), the main causes of documented extinctions over the past several centuries are habitat loss, overexploitation and introduction of alien species. Often two, or all three, combine. All three are the result—directly for the first two, and often unwittingly for the third—of human activities.

More recently, the ever-growing atmospheric input of the greenhouse gas carbon dioxide from the burning of fossil fuels is causing climate change that can amplify these existing threats, and also add new ones, both regionally and globally. Although there are doubts about the detailed time scales of some nonlinear processes (melting of glaciers and ice caps; alterations in thermohaline flows; thawing permafrost), there are no doubts that climate change is real, primarily human created and with serious consequences. The deleterious effects on many plant and non-human animal populations are already apparent (Root *et al*. 2003; Thomas *et al*. 2004; Kerr & Kharuba 2007).

**Figure 2.**
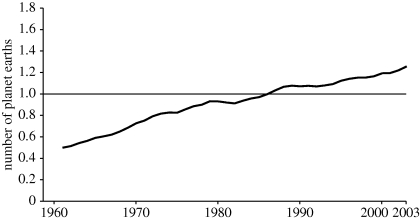
An estimate of the total EF of the human population, 1960–2001, as defined and discussed in the text. The straight line shows our planet's estimated biological capacity, that is the total EF available on a sustainable basis (after WWF 2008).

In short, the causes of the dramatic acceleration in species' extinction rates over the past century and more is unambiguously the growth in humanity's EF, itself a product of increasing human numbers and increasing impact per person. As defined by WWF (2008), the EF for a specific country in a given year measures its demand on the biosphere in terms of the area of biologically productive land and sea required to provide the resources used and to absorb the waste produced by that country, given its inhabitants' collective habits in that year. The global EF is then simply the sum over all countries. Figure 2 illustrates the trends and—recognizing the imprecisions inherent in such estimates—nevertheless suggests that we are at or beyond sustainable limits.

Some interesting paradoxes are inherent in this concept. The average Swede in 2001 had an EF of 7.0 ha, in a country where a 9.8 ha EF was sustainable. The equivalent figures for Egypt were 1.5 and 0.5 ha. Who is the more virtuous: the average Swede living within the country's sustainable limits or the average Egyptian with roughly one-fifth the personal EF yet exceeding the country's sustainable capacity by a factor of three? We will return to this point below, but first look in more detail at historical trends in overall growth of human populations and of *per capita* impacts.

## 3. Human population growth

It is a fairly common misconception that human numbers have increased roughly exponentially since *Homo sapiens* first emerged. Nothing could be further from the truth. During our roughly 200 000 years as hunter-gatherers, human numbers probably never exceeded 5–10 million. With the beginning of agriculture and early cities, some 10 000 years ago, this began to change. Populations are estimated to have grown faster in the first 5000 years of this journey than in the second (until roughly the middle of the previous millennium), owing to the impact of infectious diseases that could not be maintained in low-density populations (endemic measles, for example, needs populations of half a million or so). The scientific–industrial revolution in the West saw another surge. But, overall, it took all of human history to attain the first billion, around 1830; 100 years to double again; 40 for the next doubling, around 1970 and we are now roughly 6.7 billion. There has never been anything remotely similar to the past 60 years or so, with the advent of truly science-based medical understanding, which has seen human populations roughly treble in the span of one lifetime.

One way of emphasizing the singularity of the recent past is to note that 50 years ago the average life expectancy at birth was 46 years. Today it is 64 years. In the developed world, we may have difficulty relating to a life expectancy of 46 years only 50 years ago, but this is because the difference in life expectancy between developed and developing worlds then was 26 years; the corresponding figure today is a still disgraceful 12 years. Another way is to observe that the total number of humans ever to have lived is estimated at around (a bit less than) 100 billion. One of Walt Whitman's poems has a memorable image—thinking of all past people lined up in orderly columns behind those living—‘row upon row rise the phantoms behind us’. Actually, looking over our shoulder, we would see only around 15 rows.

In remarkable contrast to these age-old trends, the current decade—and possibly even the current year, 2009—sees a ‘tipping point’ in our demographic history: the planet's average woman is having almost exactly one female child. That is, global average fertility rates are at replacement levels. Of course, the recent past results in age profiles containing many more children and young people than older ones, so that if indeed fertility rates continue at replacement levels the population will continue to grow, to some nine billion or so, before possibly coming to equilibrium around 2050.

These relatively recent changes in demographic patterns can be brusquely but accurately summarized as resulting from increasing education and empowerment of women, and rising prosperity. The former is arguably more important than the latter, a view that is supported by the fact that such global averages also contain great disparities among individual countries. And these disparities are not simply between the developed and the developing world: the USA, for example, currently has average fertility rates well above replacement (in contrast to most European countries, many of which have declining populations), while Bangladesh, which from its beginning as an independent nation has fostered education of women and non-coercive availability of fertility control, shows fertility rates approaching replacement levels (in marked contrast with Pakistan, where fertility rates remain high). And these contrasting examples could be multiplied many times over.

Against this background, and also mindful of the Royal Society's 350th Anniversary, it is worth noting that in 1992 the Presidents of the Indian National Science Academy, the US National Academy of Sciences, the Royal Swedish Academy of Sciences and the Royal Society proposed a meeting of the world's academies of science, to prepare a joint statement about population, in preparation for the UN Conference on Population and Development in Cairo in 1994. Fifty-eight academies attended this ‘Population Summit of the World's Science Academies’ in New Delhi in 1993. This meeting placed strong emphasis on the education and empowerment of women. The meeting had one fruitful outcome, in that it paved the way for the establishment of the InterAcademy Council in 2001. But in its initial and primary purpose it was less successful. It provoked a counter-movement by a fundamentalist ‘coalition of the unwilling’, led by the religious right in the USA, the Vatican and Saudi Arabia, which essentially took women's rights off the Cairo agenda and went on to the truly remarkable achievement of removing any mention of population from the UN's Millennium Development Goals.

## 4. Humanity's ecological impacts

When we were hunter-gatherers, our energy consumption was little more than that required, and obtained from food, to maintain metabolic processes. Conforming to a rather pervasive but ill-understood ecological rule that holds for many other animals, humans spent about 0.1 of a calorie to put 1 calorie into their mouths. By around 1900, although roughly half the workforce in developed countries were still to be found on the farm (in the UK, ahead of the wave, the proportion was declining to around 35%), advances in agricultural science meant it took 1 calorie to put 1 calorie on the table. Today the ratio, in developing countries, is more like 10:1, or more. And this 100-fold energy increase compared with earlier ages is supplied mainly by burning fossil fuels.

Vitousek *et al*. (1986) estimated that humans use, directly or indirectly, approximately 40 per cent of all terrestrial net primary productivity. Subsequent analyses of satellite images confirmed this, showing 40 per cent of land area modified by humans. Global agriculture currently uses 60 per cent of all run-off water (Sachs 2008), and projecting current trends in demand (70% for agriculture) versus sustainable supply of fresh water shows the curves crossing around 2040. Note also that of all the atmospheric nitrogen fixed in 2007, 55 per cent came from the Haber–Bosch chemical process, subsidised by fossil fuels, rather than from the natural biogeochemical processes that built the biosphere.

The wider implications for feeding tomorrow's world are explored in Beddington's essay in this volume. The implications for other animals and plants, and thence for sustainability of ecosystem services, were touched upon above, but one aspect merits revisiting here. An important, but relatively underdeveloped, area of ecological science asks: how can we alter habitats and ecosystems to provide for human needs, but do so subject to constraints which preserve both particular individual species and key elements of the ecosystems? This will be no easy trick, as it involves detailed ecological understanding case by case. Terborgh's (1983) *Five new world primates* is a pioneering work in this arena. It identifies a specific subset of tree species that would need to be kept in order for more intensive human exploitation of forests in the region of his study site to be reconciled with the continued survival there of five species of New World Monkeys, all omnivores with a mixed diet of different fruits and small prey items. A more wide ranging discussion of these issues, in an African context, is given by Western *et al*. (1994). There is, in my opinion, much need for more work on this subject.

Not only agriculture, but essentially all human activities—at home, at work, in the market place—involve external energy subsidies. These amount, as a global average although with huge variations from country to country, to 15 times that required to sustain metabolic processes. Roughly 80 per cent of these energy subsidies currently come from fossil fuels (coal, oil, gas), 10 per cent from biomass (some burned on a sustainable basis, some not), 7 per cent from nuclear and 3–4% from all other renewables. That is, 80–90% is putting carbon dioxide into the atmosphere. In the WWF's calculations of EF, accounting for such greenhouse gases—particularly in terms of preserving forests as buffers—is a major source of imprecision, although these difficulties do tend to cancel out in comparisons between actual and sustainable EFs in figure 2.

**Figure 3.**
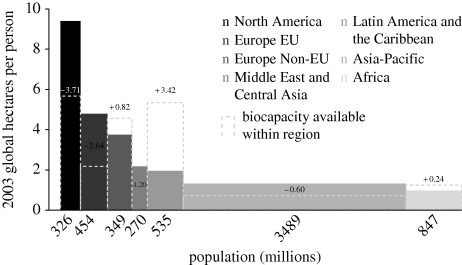
The average human EF in 2003 is shown, in units of area, for each of the planet's major geographical regions. The corresponding populations are shown on the *x*-axis, and the resulting rectangular areas represent the total ecological footprint by region. Adding all these together gives the total human ecological footprint, as shown in figure 2 (after, WWF 2008).

Figure 3 shows, for the planet's major regions, the average EF per person (the height of the respective area) and the total population (the width of the area). Obviously, the total footprint for each region is the area given by multiplying the total population times the average impact, EF, per person. The inequities are obvious, which takes us to the final section.

## 5. Policy implications for a sustainable world

The actions needed to produce a sustainable future are as obvious in principle as they are difficult in practice.

For the population side of the equation, we need continued progress in educating women and giving them control over their lives, along with access to fertility control if that is their choice. The facts outlined earlier speak clearly for the efficacy of this. They also imply much stronger international action to oppose agencies and countries that tie international aid to forbidding distribution of condoms (even in countries with high incidence of HIV).

For the *per capita* impact aspects, arguably the most important single action will be the decarbonization of energy supplies. Given that only 3–4% of energy supplies currently come from renewable sources, this will not be easy. It is, however, in principle possible, as indicated for the UK by the first report of its Committee on Climate Change (2008). Other measures involve the ‘doubly green revolution’ in agriculture (Conway 2007), as discussed in this volume by Beddington. Also important are scientific advances in understanding how to reconcile human uses of land and sea with preservation of ecosystem services and their attendant species, as called for above. The benefits of avoiding unnecessarily wasteful practices, and of adapting to (rather than resisting) some forms of environmental or cultural change, also should not be underestimated.

These huge and global problems—climate change, loss of biological diversity, pressure on water supplies and much else—demand globally cooperative solutions. The problems are further compounded by the need for nations to cooperate, but to cooperate in equitable proportions.

One glance at figure 3 makes obvious the need for action to be taken in a way which is equitably proportioned among countries. The rich world, with 13 per cent of the global population, has more than half the global GDP and likewise consumes roughly half the energy and generates roughly half the greenhouse gas inputs. Even more, it is responsible for roughly 80 per cent of the greenhouse gases already added to the atmosphere, most of which will remain there for decades to come.

These questions of cooperation, when ‘I will if you will’ too easily can degenerate into ‘I won't if you won't’, are explored in more detail in this volume by Levin and (rather differently) by Nowak. The essential point is that, although all would be better off if all cooperated, such systems are vulnerable to ‘cheats’, who enjoy the collective benefits without paying their dues. Such problems are receiving increasing attention in the scholarly literature, employing a variety of metaphors: the Tragedy of the Commons; the Free-Rider problem; the Prisoner's Dilemma and others. These metaphors are allied to artificial games in which the subjects (usually undergraduates) trade small sums of money to test limits to altruism and tolerance of cheating. Importantly, essentially none of this work involves the costs and benefits varying among the players, as it certainly does in the real world of figure 3.

My own speculation about how cooperative human societies evolved is both less academic and analytic, and more gloomy. Once we move out of the mists of pre-history, we find stories of dreamtime, creation myths, ceremonies and initiation rites, spirits and gods, with a unifying theme that all seek simultaneously to help explain the external world and also to provide a ‘stabilization matrix’ for a cohesive society. There are, moreover, some striking and unexplained similarities in belief systems and rituals from different times and places. Conscience, a simple word for a complex concept that helps foster behaviour in accord with society's professed norms, has been memorably defined by H. L. Mencken as ‘the inner voice which warns us that somebody might be looking’. And how helpful it is if that somebody is an all-seeing, all-knowing supernatural entity.

Common to these conjectured ‘stabilizing forces’ in essentially all earlier societies are hierarchical structures, serving and interpreting the divine being or pantheon, along with unquestioning respect for authority. In such systems, faith trumps evidence.

But if indeed this is broadly the explanation for how cooperative behaviour has evolved and been maintained in human society, it could be bad news. Because although such authoritarian systems seem to be good at preserving social coherence and an orderly society, they are, by the same token, not good at adapting to change (Diamond 2005).

A fundamental principle emerging from the Neo-Darwinian Synthesis of around a century ago is Fisher's Fundamental Theorem, which states that a population's potential rate of change of gene frequency (which measures its ability to adapt to changing circumstances) is proportional to the variance in gene frequency, which will be small if essentially all individuals are well adapted to their current environment. That is, there is an inherent tension between adaptedness and adaptability. If there is any substance in my speculations about the answer to Darwin's problem in explaining cooperation in human societies, we again have a fundamental tension—at the level of the entire society—between, on the one hand, ‘ties that bind’ and permit stably cooperative aggregations, and on the other hand, ability to respond effectively to changing environmental circumstances. It could even be argued that the recent rise of fundamentalism, in both East and West, is an illustration of this meta-level version of Fisher's Fundamental Theorem, as complex faiths are reduced to intolerant ideologies to resist the challenge of societal change. I hope my thoughts on this question, probably the most challenging question in evolutionary biology, are wrong.
